# Network Pharmacological Study on the Mechanism of Cynanchum paniculatum (Xuchangqing) in the Treatment of Bungarus multicinctus Bites

**DOI:** 10.1155/2022/3887072

**Published:** 2022-07-05

**Authors:** Linsheng Zeng, Jingjing Hou, Cuihong Ge, Yanjun Li, Jianhua Gao, Congcong Zhang, Chengbin Li, Yuxiang Liu, Zhongyi Zeng

**Affiliations:** ^1^Department of Emergency, Shenzhen Traditional Chinese Medicine Hospital, Shenzhen, China; ^2^Department of Emergency, The Fourth Clinical Medical College of Guangzhou University of Chinese Medicine, Shenzhen, China

## Abstract

**Background:**

Bungarus multicinctus is one of the top ten venomous snakes in China. Its venom is mainly neurotoxin-based. Novel antivenom drugs need to be further researched and developed.

**Objective:**

This study aimed to explore the molecular mechanism of Cynanchum paniculatum in treating Bungarus multicinctus bites based on network pharmacology. *Material and methods*. The potential active ingredients of Cynanchum paniculatum were screened and their SDF structures were obtained using the PubChem database and imported into the SwissTargetPrediction database, and targets were obtained for the antitoxin effects of Cynanchum paniculatum in the treatment of Bungarus multicinctus bites. The Cynanchum paniculatum-active compound-potential target network and protein-protein interaction network were constructed by using Cytoscape software, and then biological function analysis and KEGG pathway enrichment analysis were performed using the DAVID.

**Results:**

Seven potential active components (cynapanoside C, cynatratoside B, tomentolide A, sitosterol, sarcostin, tomentogenin, and paeonol) and 286 drug targets were obtained, including 30 key targets for the treatment of bungarotoxin toxicity. The active components mainly acted on PIK3CA, MAPK1, MAP2K1, JAK2, FYN, ACHE, CHRNA7, CHRNA4, and CHRNB2, and they antagonized the inhibitory effect of bungarotoxin on the nervous system through cholinergic synapses and the neurotrophin signaling pathway.

**Conclusions:**

Cynanchum paniculatum exerts a therapeutic effect on Bungarus multicinctus bites through multiple active components, multiple targets, and multiple pathways. The findings provide a theoretical basis for the extraction of active components of Cynanchum paniculatum and for related antivenom experiments.

## 1. Background

Snakebite is a neglected tropical disease [1]. It is estimated that about 5 million people worldwide are bitten by snakes each year, of which 81,000 to 138,000 die, and many people have permanent physical and psychological sequelae [2]. Venomous snake bites are a constant threat to human health and life. Bungarus multicinctus is one of the top ten venomous snakes in China. Its venom is mainly neurotoxin-based [3], containing *α*-, *β*-, and *γ*-bungarotoxins (BGTs) [4, 5]. Bungarus multicinctus bites cause neuromuscular symptoms, including ptosis, mydriasis, ophthalmoplegia, mandibular weakness, neck muscle paralysis, limb paralysis, respiratory muscle paralysis, respiratory failure, and even death [6, 7]. Therefore, the timely treatment of Bungarus multicinctus bites is very important. Antivenom is currently the most effective and commonly used treatment for Bungarus multicinctus bites. According to the WHO, because it is expensive and difficult to store, antivenom is challenging to obtain in economically disadvantaged and remote rural areas, and there is still great risk associated with antivenom treatment [8]. In China, horse-derived antivenom is currently used in clinical, but the allergic reaction rate can be high, ranging from 3% to 88% [9]. Therefore, novel antivenom drugs need to be further researched and developed.

Network pharmacology is an emerging discipline based on systems biology [10, 11]. It involves integration of multiple disciplines, such as high-throughput omics, computer technology, pharmacology, and network database retrieval, and can be used to reveal the pharmacological effects of traditional Chinese medicines and their molecular mechanisms [12]. Network pharmacology has been demonstrated to be a powerful method for analysis of the underlying mechanism of Chinese medicine ingredients [13]. Traditional Chinese medicines contain many active ingredients, including multiple ingredients that can act on multiple cellular targets and pathways in different ways [14]. Network pharmacology is particularly well-suited for analyses of the characteristics of these multiple targets and multiple pathways and can improve the success rates of clinical trials on new drugs and reduce the cost of drug research [15].

It is also applicable for the research methods of network pharmacology to the study of toxicology. Xiaohui Fan et al. have proposed the concepts of network toxicology and network toxicology of traditional Chinese medicine [16]. And firstly extracted the key elements: gene, protein, toxicity, side effects, and others from the database; then took these elements as nodes in the network to construct a network model, which included network of interactions among gene, target, and drug interaction. Through this network analysis, we can understand the relevant toxic mechanism and find out the effective components of treatment or substances with toxic and side effects. Until now, there are many studies that have been successfully carried out by many researchers through network toxicology, such as Haonan Ruan et al. reported that they had predicted some targets of Mycotoxin-Induced Liver Injury (MILI) through network toxicology, which provided a theoretical basis for further study of the toxicity mechanism [17]. Also, Yubo Li et al. published that they had preliminarily identified the toxic compounds of Chinese medicine and gave a comprehensive explanation of its toxic mechanism by the prediction results of network toxicology [18]; moreover, Tao He et al. have explored and found the mechanism of hepatotoxicity induced by Esculent side A in rats and analyzed the changes of endogenous metabolites in rat plasma through combining the network toxicology with nontargeted metabolomics [19]. Therefore, in this study, we employed the approach of network pharmacological and toxicological again to investigate the toxicity mechanism of bungarotoxin and the therapeutic target sites of Cynanchum paniculatum.

Modern pharmacology shows that the various active ingredients of Cynanchum paniculatum can treat various diseases [20]. Extensive pharmacological activities of the extracts or compounds of Cynanchum paniculatum in vivo and in vitro were confirmed, which included the effect of anti-inflammatory, antinociceptive, sedative antiviral, antitumor, neuroprotective, treating snakebites, immunomodulatory, antiradiation, vasodilatory, acaricidal potentials, and antiadipogenic [21]: Paeonol is one of the simple phenolic compounds, which can be extracted from Cynanchum paniculatum and has various biological and pharmacological activities, such as anti-inflammatory, antitumor, antihypertensive, and antioxidant [22–24]. Panying Wei et al. also found that two compounds which were isolated from Cynanchum paniculatum and elucidated as cynanversicoside A and cynanversicoside C showed much strong anti-inflammatory and antiviral activities [25]. Moreover, Zhao Dan et al. reported that C21 steroidal glycosides obtained from the roots of Cynanchum paniculatum had the function of antioxidant and antibacterial [26]. Wen Ji-Hong et al. have found that there were two extracts from Cynanchum paniculatum that had the activity against Ichthyophthirius multifiliis theronts and tomonts [27]. Moreover, Antofine which is extracted from Cynanchum paniculatum is also a phenanthroindolizidine alkaloid with antiproliferative and antitumor effects [28]. Jin Bae Weon et al. reported that they successfully isolated the neuroprotective compounds which may antagonize the neurological damage caused by bungarotoxin from Cynanchum paniculatum [29]. Yan Xiong et al. also published that the ethanolic root extract of Cynanchum paniculatum had the properties of anti-deinagkistrodon acutus venom [30].

Chinese medicines have been used to treat venomous snake bites for thousands of years, many of them with good clinical efficacy. Among them is Cynanchum paniculatum, also known as the “snake dysentery herb,” which is particularly effective against Bungarus multicinctus bites. Modern pharmacology has confirmed that Cynanchum paniculatum extract contains active antisnake venom ingredients [30, 31]. Intragastric administration of Cynanchum paniculatum extract significantly attenuates the toxic effects of snake venom in mice [32]. In vitro enzyme activity inhibition experiments and in vivo animal protection experiments have also found that Cynanchum paniculatum has good antisnake venom activity [33]. However, Cynanchum paniculatum contains many different chemical ingredients; the specific ingredients that exert antivenom effects and their mechanisms are still unclear. Therefore, the aim of this study was to clarify Cynanchum paniculatum's active antisnake venom ingredients and their targets using network pharmacology. The findings of this study will be of great significance for the discovery and extraction of new antisnake venom compounds [34].

## 2. Material and Methods

### 2.1. Chemical Composition and Target Prediction of Cynanchum Paniculatum

The Traditional Chinese Medicine Systemic Pharmacology Database and Analysis Platform (TCMSP) (http://lsp.nwu.edu.cn/tcmsp.php/), which contains 499 herbal medicines registered in the Chinese Pharmacopoeia and their 29,384 ingredients, 3311 targets, and 837 related diseases, was used to predict the chemical composition of Cynanchum paniculatum [35]. An oral bioavailability (OB) ≥30% and drug-likeness (DL) ≥0.18 were used as screening conditions, and the literature was used to supplement the potential active ingredients. Afterward, the structure data file (SDF) structures of the above ingredients were obtained using the PubChem database [36, 37] (https://pubchem.ncbi.nlm.nih.gov/) and then imported into the SwissTargetPrediction [38] (http://www.swisstargetprediction.ch/) database. Based on the 2D and 3D similarities of the chemical components with human as the selected species of “human,” the potential targets of the compounds were predicted, and the potential targets of Cynanchum paniculatum were ultimately obtained by collating the results and removing duplicates.

### 2.2. Acquisition of Targets for BGT

The Online Mendelian Inheritance in Man (OMIM) [39] (https://omim.org/) and GeneCards [40, 41] (https://www.genecards.org/) databases were searched with “bungarotoxin” as the keyword to obtain the targets of BGT.

### 2.3. Construction of a Cynanchum Paniculatum-Active Compound-Potential Target Network

The potential targets of Cynanchum paniculatum were mapped to the targets of BGT to obtain the potential targets of Cynanchum paniculatum in the treatment of BGT toxicity. The active compounds and potential targets obtained above were introduced into Cytoscape [42, 43] software (Version 3.6.1, http://www.cytoscape.org) to draw a Cynanchum paniculatum-active compound-potential target network. In this network, the different types of nodes represent Cynanchum paniculatum, its active compounds, and its potential targets, and the relationships among them are shown as edges. The Network Analyzer [44] plug-in was used to analyze the main active ingredients of Cynanchum paniculatum with the “betweenness centrality” and “degree” settings (the thickness of each edge reflects the magnitude of the betweenness centrality, and the size of each node reflects the magnitude of the degree. A larger degree value is associated with a more important component).

### 2.4. Protein-Protein Interaction (PPI) Network Construction

A PPI network was constructed by inputting the common targets of Cynanchum paniculatum and BGT into the STRING database [45] with the species set as “Homo sapiens” and the minimum interaction threshold set as 0.4. The target interaction of network data was imported into Cytoscape software to draw the protein interaction network and analyze the topology of the network. The color and size of the network nodes were set with the “Generate style from statistics” tool. The size, color, and shade of the nodes reflect the magnitude of the degree value, and the thickness of each edge indicates the magnitude of the combined score.

### 2.5. Gene Ontology (GO) Analysis and Kyoto Encyclopedia of Genes and Genomes (KEGG) Pathway Enrichment Analysis

The Bioconductor [46] package “(http://org.Hs.eg.db/)” was installed in R software and run to convert Cynanchum paniculatum/BGT targets and common targets into entrezIDs. The “ClusterProfiler” package was installed in R software, and GO and KEGG functional enrichment analyses of the key target genes were performed with thresholds of a *P* < 0.05 and a *Q* < 0.05 based on the converted entrezIDs. The results were output in the form of bar graphs.

## 3. Results

### 3.1. Screening of Active Ingredients and Targets of Cynanchum Paniculatum

The active ingredients of Cynanchum paniculatum in the TCMSP database were screened with the settings OB ≥30% and DL ≥0.18, and paeonol was included according to the literature [47, 48] to obtain a total of seven potential active ingredients: cynapanoside C, cynatratoside B, tomentolide A, sitosterol, sarcostin, tomentogenin, and paeonol. The SDF structures of the above components were obtained using the PubChem database and imported into the SwissTargetPrediction database, and a total of 286 drug targets were screened after deduplication.

### 3.2. Prediction of the Targets of Action of BGT

**Figure 1:**
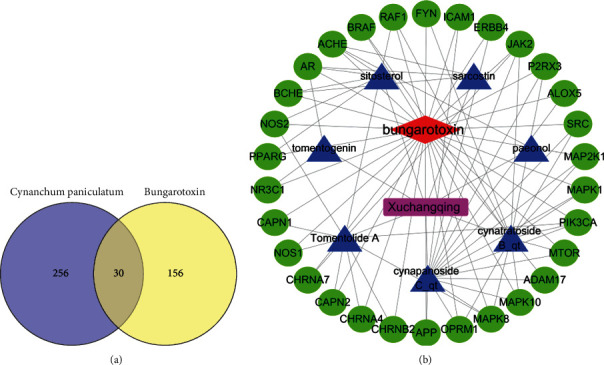
Venn diagram and network diagram for describing the interaction. (a) Venn diagram of the overlapping targets of Cynanchum paniculatum and Bungarotoxin. (b) A network diagram of Cynanchum paniculatum component-bungarotoxin target interactions.

A total of 186 disease targets of BGT were obtained after deduplication using the OMIM and GeneCards databases. A total of 286 drug targets and 186 disease targets were entered into the Venny2.1 online mapping tool to create a Venn diagram, and 30 common targets were obtained after the intersection of the drug and disease targets (Figure 1(a)). The 30 common drug-disease targets included APP, PIK3CA, JAK2, ACHE, CAPN1, OPRM1, MAPK1, ERBB4, AR, NOS1, MAPK8, MAP2K1, ICAM1, BCHE, CHRNA7, MAPK10, SRC, FYN, NOS2, CAPN2, ADAM17, ALOX5, RAF1, PPARG, CHRNA4, MTOR, P2RX3, BRAF, NR3C1, and CHRNB2.

### 3.3. Construction and Analysis of the Cynanchum Paniculatum Component BGT Target Interaction Network

The seven potential active ingredients and 30 common drug-disease targets for Cynanchum paniculatum were entered into Cytoscape software to construct a drug-ingredient-target-disease interaction network [49] (Figure 1(b)). In Figure 1(b), purple represents the drug, blue represents the 7 active ingredients in Cynanchum paniculatum, green represents the 30 common targets, and red represents the disease. The degrees of freedom are listed in descending order, as follows: cynapanoside C, 16; cynatratoside B, 14; tomentolide A, 12; sitosterol, 6; sarcostin, 4; tomentogenin, 3; and paeonol, 2. Topological analysis of the 30 common targets revealed that the targets with ≥4 degrees of freedom were MAPK8, PIK3CA, JAK2, ACHE, and AR. It is evident that the targets of Cynanchum paniculatum in the treatment of Bungarus multicinctus bites are diversified and act as antitoxins through synergistic effects on multiple targets. Paeonol was the most abundant component in Cynanchum paniculatum, but there was only one common target with the disease (ACHE), suggesting that paeonol may act as a therapeutic agent by affecting ACHE.

### 3.4. PPI Network Construction and Core Target Analysis

#### 3.4.1. PPI Data Construction

**Figure 2:**
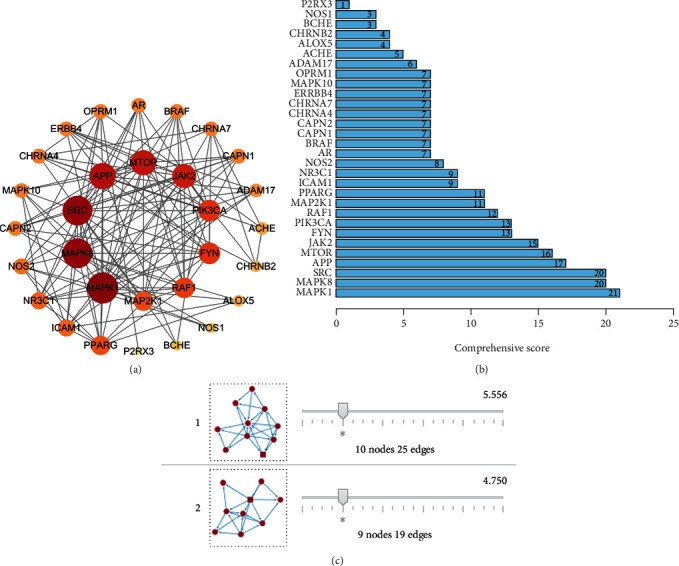
Targets screening. (a) The network diagram of PPI. (b) Ranking score of core targets by topology analysis. (c) MCODE Gene cluster analysis.

The common targets of Cynanchum paniculatum/BGT were inputted into the STRING database to obtain the target network relationship data, which were then imported into Cytoscape 3.7.2 to draw the protein interaction network diagram (Figure 2(a)). MAPK1, SRC, and MAPK8 had degrees greater than or equal to 20.

#### 3.4.2. Core Target Screening Based on Topology Analysis

The PPI network was imported into Cytoscape 3.7.2, and the topology analysis was carried out with Network Analyzer. The four parameters of degree, betweenness centrality, and average shortest path length and closeness centrality were used as reference standards, and the genes with scores greater than the average score were selected as the core targets. The top 30 targets were plotted in a bar graph using R3.6.0 (Figure 2(b)).

#### 3.4.3. Core Target Screening Based on Cluster Analysis

The constructed PPI network was introduced into Cytoscape 3.7.2, and the MCODE module was used to analyze gene clusters and screen core targets. Two gene clusters (Figure 2(c)) and two core genes (nuclear receptor subfamily 3, group C, member 1 (NR3C1) and MAPK8) were obtained. It is speculated that the effective components of Cynanchum paniculatum may play a major therapeutic role through NR3C1 and MAPK8.

#### 3.4.4. GO Analysis

**Figure 3:**
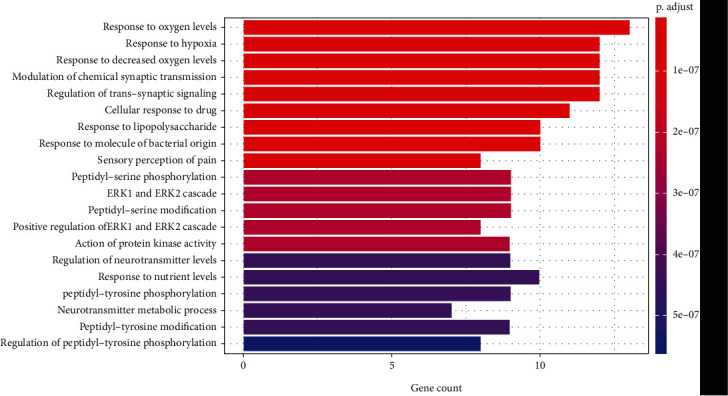
Biological processes associated with the 30 common targets, as determined by GO analysis.

**Figure 4:**
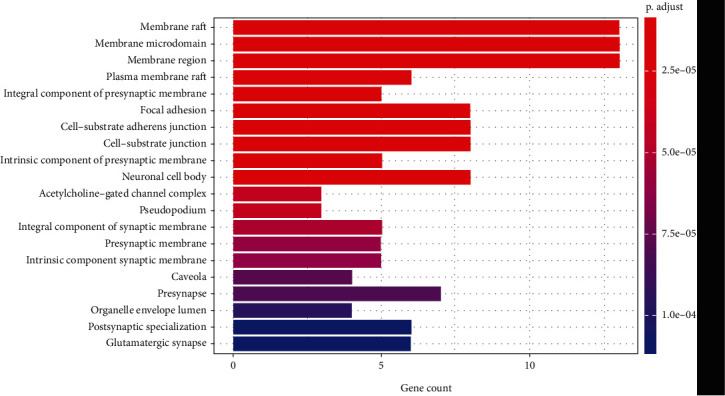
Cellular components associated with the 30 common targets, as determined by GO analysis.

**Figure 5:**
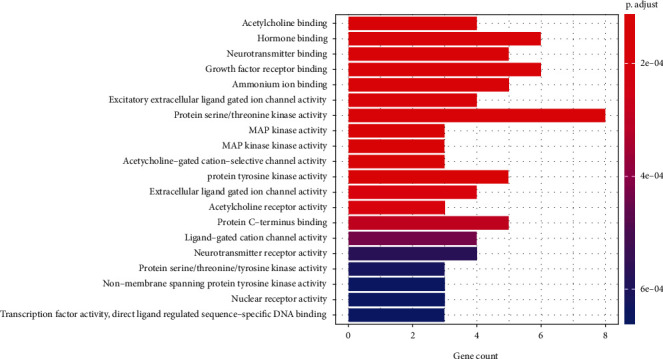
Molecular functions associated with the 30 common targets, as determined by GO analysis.

The 30 common targets were subjected to GO analysis for the biological process, cellular component, and molecular function categories. GO analysis showed that the intersecting genes were enriched for a total of 868 biological process terms, mainly including the hypoxic response, regulation of chemical synaptic transmission, glucose metabolism, drug metabolism, antimicrobial action, regulation of trans-synaptic signaling, and neurotransmitter metabolic process terms (Figure 3). The intersecting genes were enriched for 76 cellular component terms, mainly the cytoplasm, cell membrane, presynaptic membrane, cell-basal node, inherent components of presynaptic membrane, and inherent components of the presynaptic membrane terms (Figure 4). The intersecting genes were enriched for a total of 78 terms related to molecular function, mainly the acetylcholine binding, hormone binding, neurotransmitter binding, growth factor receptor binding, ammonium ion binding, excitatory extracellular ligand-gated ion channel activity, protein serine/threonine kinase activity, MAP kinase activity, and acetylcholine-gated cation-selective channel activity terms (Figure 5).

#### 3.4.5. KEGG Pathway Enrichment Analysis

**Figure 6:**
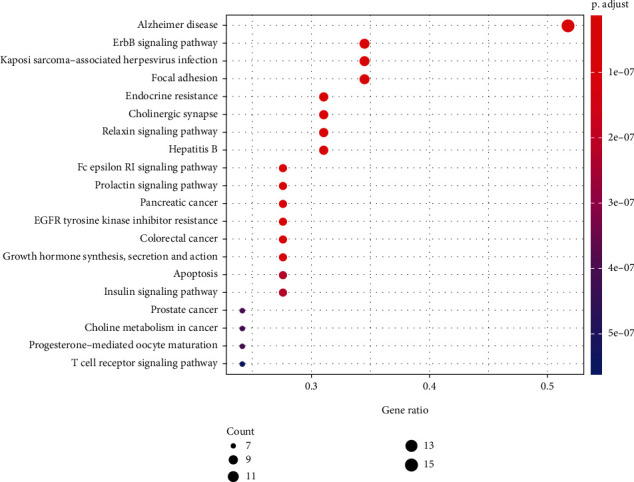
KEGG enrichment analysis of the pathways associated with bungarotoxin treatment using Cynanchum paniculatum.

A total of 133 KEGG pathways were obtained by running the 30 common targets through R software. The top 20 pathways were used to create a bubble plot of KEGG functional enrichment (Figure 6), with the *P* value representing the significance of the enrichment and a redder color indicating greater significance. The ERbB signaling, Alzheimer's disease, endocrine resistance, Fc epsilon RI signaling, prolactin signaling, and cholinergic synapse pathways had the highest relevance; the cholinergic synapse signaling pathway is the most clinically relevant pathway and may play key roles in many of the other signaling pathways. The red dots in the cholinergic synaptic signaling pathway are important links of the common targets involved in the process. The main targets involved are PIK3CA, MAPK1, MAP2K1, JAK2, FYN, ACHE, CHRNA7, CHRNA4, and CHRNB2.

## 4. Discussion

Chinese herbs consist of multiple compounds with complex mechanisms of action that may affect multiple targets and pathways in humans [50]. Cynanchum paniculatum is a Chinese herb used to treat venomous snake bites with good clinical efficacy, but its therapeutic mechanism is still not well known. In this study, a network pharmacology approach was used to screen the active compounds against BGT and to analyze the network pathways and therapeutic mechanisms of this herb using multiple databases. All the chemical constituents contained in Cynanchum paniculatum were screened by using the TCMSP database. According to the results combined with previous literature reports, 7 compounds with good OB and DL properties were selected. After further screening using the SwissTargetPrediction database, 286 drug targets and 30 common drug-disease targets were obtained. Topological analysis of the 30 common targets revealed that MAPK8, PIK3CA, JAK2, ACHE, and AR were the most likely targets through which Cynanchum paniculatum antagonizes BGT. Given the clinical symptoms of BGT toxicity, ACHE may be the most important target. Network analysis (Figure 2) showed that paeonol exerts antagonistic effects by interacting with ACHE. Similarly, it has been demonstrated that paeonol also has efficacy against Agkistrodon toxin [33].

PPI analysis and topology analysis revealed that MAPK1, SRC, and MAPK8 were the most important core targets, while cynapanoside C and cynatratoside B were the active ingredients corresponding to the three core targets. MAPK is an important transmitter of signals from the surface of the cell to the inside of the nucleus and can regulate a variety of important cellular physiological and pathological processes, such as the stress response and inflammatory response. Studies have suggested that MAPK1 is an important anti-inflammatory and antivenom target [51]. Hossen et al. pointed out that Persicaria chinensis L. might play a pivotal ethnopharmacologic role as an anti-inflammatory herbal medicine by targeting Syk and Src kinases and their downstream transcription factor NF-*κ*B [52]. Another study has reported that inhibiting the MAPK8/ERK signaling pathway can protect H9C2 cells from oxidative stress damage [53]. Additionally, gene cluster analysis screened out two core genes, NR3C1 and MAPK8, that corresponded to tomentolide A, cynapanoside C, and cynatratoside B. Mylka et al. found that NR3C1 contributes to effective anti-inflammatory therapy [54]. In summary, these findings indicated that MAPK1, SRC, NR3C1, and MAPK8 may be important targets that antagonize the effects of BGT.

GO analysis showed that the main cellular components involved in the biological processes of chemical synaptic transmission modulation, trans-synaptic signaling regulation, and neurotransmitter metabolism are the presynaptic membranes and posterior membranes of nerve cells. The corresponding molecules that perform these biological functions include receptors on the presynaptic membranes and posterior membranes of nerve cells and various excitatory or inhibitory neurotransmitters, among which MAP kinase and acetylcholine are the main ones. BGT contains very potent neurotoxins [55] and is a heterodimer containing two different subunits (*α*-BGT and *β*-BGT) [56]. *α*-BGT binds to motor endplate acetylcholine receptors and then dissociates very slowly from the receptors. In contrast, *β*-BGT acts on the presynaptic membrane, first inhibiting the release of neurotransmitters and then promoting the release of neurotransmitters, both of which act together to cause persistent muscle paralysis [57, 58]. Clinically, a bungarus multicinctus bite can cause nerve paralysis, such as limb numbness and weakness, drooping eyelids, and respiratory muscle paralysis. The results of GO analysis are consistent with clinical findings and with modern toxicological and pharmacological research.

KEGG pathway enrichment analysis and clinical studies have shown that the cholinergic synaptic pathway plays key roles in many signaling pathways. ACHE, the most important target in the signaling pathway, plays important roles in the release and transmission of neurotransmitters. Figure 2(b) shows that sarcostin, paeonol, and ACHE are the common (overlapping) targets. The mechanism of action may involve blockade of the binding of bungarotoxin to ACHE; alternatively, sarcostin and paeonol may directly bind to bungarotoxin to change its structure and prevent it from binding to the ACHE receptor, thereby exerting a detoxification effect. Modern pharmacological studies have indicated that paeonol has a wide range of pharmacological activities, including anti-inflammatory, antioxidant, antipyretic, analgesic, neuroprotective, anticancer, and antiviral activities [23, 59, 60]. Han et al. found that paeonol inhibited the reduction and degeneration of dendritic spines in the frontal cortex of the brain in a rat model of Alzheimer's disease [61]. Miao et al. found that paeonol alleviated inflammation in the nucleus by restricting HMGB1 [62]. Guo et al. found that paeonol protected melanocytes from H_2_O_2_-induced oxidative stress through an Nrf2-mediated antioxidant pathway [63]. These experimental findings on anti-inflammatory, antioxidant, and nerve cell protection also support the evidence obtained in this network pharmacodynamic analysis.

At present, the main drug for the treatment of Bungarus multicinctus bites is anti-Bungarus multicinctus bites venom serum. Xu Changqing, as an important adjuvant therapy drug, also plays an important role in clinical treatment. However, the compounds contained in Xu Changqing are much more complex, and until now, it has not been confirmed which chemical components play the key role of antitoxin. Therefore, this study analyzes its possible mechanism of action through the analysis method of network pharmacology. However, the data of the article through network pharmacology analysis is theoretically credible, which still needs to be confirmed by further animal experiments.

Until now, there have been many research reports in vivo and in vitro that have confirmed that Cynanchum paniculatum has extensive antivenom effects [21, 30]. And the clinical efficacy of Cynanchum paniculatum in the treating cobra bites and the modern pharmacological evidence of bungarotoxin has corroborated the results of our study: firstly, modern toxicology has proved that the toxin of bungalow mainly exerts its toxic effect by blocking the conduction of the nerve-muscle junction [64–66]; so the poisoned patients can have symptoms of muscle paralysis. However, in the actual treatment, it was found that Cynanchum paniculatum can alleviate these clinical symptoms, which can be seen that the action site of Cynanchum paniculatum's treatment is also located at the nerve-muscle junction [67, 68]. It mainly includes neurotransmitters secreted by the presynaptic membrane and acetylcholine receptors on the motor end plate of the postsynaptic membrane; secondly, the results of network pharmacology analysis showed that Cynanchum paniculatum may play a therapeutic role mainly through the cholinergic synaptic signaling pathway and ACHE targets. From these points, it can be seen that the two views are mutually confirmed.

## 5. Conclusion

In conclusion, network pharmacology revealed that the anti-bungarotoxin effects of Cynanchum paniculatum may occur through multiple components, multiple targets, and multiple signaling pathways. Among the components, paeonol is the most important and mainly acts on ACHE in the cholinergic synaptic signaling pathway. The mechanism of the anti-bungarotoxin effects of paeonol may involve blockade of the binding of *α*-BGT to motor endplate acetylcholine receptors or interference with the action of *β*-BGT on the presynaptic membranes of motor nerves. This study reveals, for the first time, the molecular mechanism of the treatment of snakebite with Cynanchum paniculatum. The focus of this study is to emphasize the role and contribution of network pharmacology in the field of snakebite research. The analysis results and clinical observations of network pharmacology support that the cholinergic synaptic signaling pathway and the ACHE target are the main therapeutic action points of Cynanchum paniculatum, which is an important pathway and target for the treatment of Bungalow snake bites. The results of this study can be used as an important direction and target for the development of new drugs against cobra toxins; however, this is only the starting step in the research and development of new drugs, and then our research group will conduct in-depth research on molecular mechanisms in vitro and in vivo. The ultimate goal is to successfully screen out high-titer antivenoms from Cynanchum paniculatum's compounds for the development of new drugs; however, this process not only takes a long time to complete but also requires a lot of financial support. Therefore, more researchers are required to work together, which can advance the research process faster. Therefore, the publication of this study may provide a direction for scientific researchers engaged in the research field of snake bite, and provide certain clues for the development of new anti-Bungarus toxin drugs.

## Data Availability

The data used to support the findings of this study are included within the article.

## Abbreviation

PPI: Protein-protein interaction
PIK3CA: Phosphatidylinositol-4,5-bisphosphate 3-kinase catalytic subunit alpha
MAPK: Mitogen-activated protein kinase
MAP2K1: Mitogen-activated protein kinase 1
JAK2: Janus kinase 2.
FYN: FYN proto-oncogene Src family tyrosine kinase.
ACHE: Acetylcholinesterase (cartwright blood group)
CHRNA7: Cholinergic receptor nicotinic alpha 7 subunit
CHRNA4: Cholinergic receptor nicotinic alpha 4 subunit
CHRNB2: Cholinergic receptor nicotinic beta 2 subunit
HMGB1: High mobility group box 1
AR: Androgen receptor
SRC: SRC proto-oncogene, nonreceptor tyrosine kinase
NR3C1: Nuclear receptor subfamily 3 group C member 1
PLA2: Phospholipase A2
BGT: Bungarotoxin
GO: Gene ontology
KEGG: Kyoto encyclopedia of genes and genomes
OB: Oral bioavailability
DL: Drug-likeness
TCMSP: Traditional Chinese medicine systems pharmacology database and analysis platform
WHO: World Health Organization.
